# When one cannot see the forest for the trees: femoral d*P*/d*t*_max_, LVEF and pulse pressure in critically ill patients

**DOI:** 10.1186/s13613-019-0586-8

**Published:** 2019-10-02

**Authors:** S. Vaquer, D. Chemla, X. Monnet

**Affiliations:** 1 Servei de Medicina Intensiva, Centre de Crítics, Corporació Sanitària Universitària Parc Taulí, Parc Taulí 1, 08208 Sabadell, Spain; 2 Departament de Medicina, Facultat de Medicina, Universitat Autònoma de Barcelona, Passeig de la Vall d´Hebron 119, 08035 Barcelona, Spain; 3 Service d’explorations fonctionnelles multidisciplinaires bi-site Béclère-Bicêtre, AP-HP, Hôpitaux universitaires Paris-Sud, 78, rue du Général Leclerc, 94270 Le Kremlin-Bicêtre, France; 4 INSERM-UMR_S999 LabEx – LERMIT, Hôpital Marie-Lannelongue, 92350 Le Plessis-Robinson, France; 5 Service de médecine intensive - réanimation, Hôpital Bicêtre, AP-HP, Hôpitaux universitaires Paris-Sud, 78, rue du Général Leclerc, 94270 Le Kremlin-Bicêtre, France

## Dear Editor,

Whether the peak rate of pressure rise (d*P*/d*t*_max_) in peripheral arteries is influenced by left ventricular (LV) contractility or by loading conditions remains controversial. We, therefore, appreciate the letter by Monge-García et al. [1] and have read it with interest.

Our colleagues challenge the use of LV ejection fraction (LVEF) as a marker of LV systolic function and its comparison with femoral d*P*/d*t*_max_ to assess the reliability of the femoral d*P*/d*t*_max_ to track changes in LV contractility [1]. We entirely agree that LVEF is not a pure marker of LV contractility, as widely documented by others [2], and as clearly acknowledged in the discussion section of our article [3]. Furthermore, we agree that the invasive measure, LV end-systolic elastance (Ees), is the gold standard methodology to estimate LV contractility, but we were unable to measure it for ethical and technical reasons. In our paper [3], we presented LVEF data, as is commonly done in similar studies, but in contrast to the inference of our colleagues’ letter [1], our conclusion was not based upon LVEF results.

In our study [3], we measured femoral d*P*/d*t*_max_ by pulse contour analysis before and after varying, LV systolic function (dobutamine infusion), preload (volume expansion and passive leg-raising) and afterload (norepinephrine) in 19 critically ill patients with cardiovascular failure [3]. Femoral d*P*/d*t*_max_ changed, not only in response to dobutamine infusion, but also following changes in cardiac loading, particularly following changes in afterload induced by variations in norepinephrine dose. In addition, changes in femoral d*P*/d*t*_max_ were strongly related to changes in arterial pulse pressure (PP) in each intervention and when all interventions were pooled (each *R* > 0.90). Interestingly, Sharman et al. have also documented strong associations between radial d*P*/d*t*_max_ and brachial PP in various populations of non-critically ill patients [4, 5]. Since PP is mainly influenced by arterial stiffness and stroke volume, and given the fact that PP explained > 80% of the variance of femoral d*P*/d*t*_max_, we concluded that femoral d*P*/d*t*_max_ was mainly influenced by pulsatile arterial load [3]. These findings suggest that femoral d*P*/d*t*_max_ is highly sensitive to changes in loading conditions, especially afterload, and thus may not be directly interchangeable with LV contractility.

In previous work [6], our colleagues have documented a linear relationship between Ees and femoral d*P*/d*t*_max_ in 10 anesthetized pigs during different loading and contractile conditions. However, while the authors present good concordance results, the strength of this relationship was rather moderate (*R*^2^ = 0.33) [6]. To our knowledge, the only other experimental study documenting the link between contractility (Ees) and femoral d*P*/d*t*_max_ is Morimont et al. [7] who studied 6 anesthetized and mechanically ventilated pigs after endotoxin-induced shock and norepinephrine infusion. In their work, authors reported that Ees and femoral d*P*/d*t*_max_ were significantly, albeit weakly correlated (*R* = 0.51, i.e., *R*^2^ = 0.26). Therefore, two independent experimental studies have documented that factors other than LV contractility (Ees) explain the vast majority (67% to 74%) of the variance of femoral d*P*/d*t*_max_ in response to LV contractility and ventricular loading changes [6, 7].

In conclusion, our results showed that changes in femoral d*P*/d*t*_max_ are essentially analogous to changes in femoral PP and therefore subject to the same dependence to variations in cardiac loading conditions, especially pulsatile arterial load. Our hypothesis is consistent with the previous results obtained at the upper arm level in non-critically ill patients [4, 5] and with results from two independent experimental studies, having failed to demonstrate strong correlation between LV contractility (Ees) and femoral d*P*/d*t*_max_ in various experimental settings [6, 7].

## Data Availability

The datasets used and/or analyzed during the original study are available from the corresponding author upon request.
